# Developmental Dynamic Dysphasia: Are Bilateral Brain Abnormalities a Signature of Inefficient Neural Plasticity?

**DOI:** 10.3389/fnhum.2020.00073

**Published:** 2020-03-24

**Authors:** Marcelo L. Berthier, Guadalupe Dávila, María José Torres-Prioris, Ignacio Moreno-Torres, Jordi Clarimón, Oriol Dols-Icardo, María J. Postigo, Victoria Fernández, Lisa Edelkraut, Lorena Moreno-Campos, Diana Molina-Sánchez, Paloma Solo de Zaldivar, Diana López-Barroso

**Affiliations:** ^1^Cognitive Neurology and Aphasia Unit, Centro de Investigaciones Médico-Sanitarias, Instituto de Investigación Biomédica de Málaga (IBIMA), University of Malaga, Málaga, Spain; ^2^Department of Psychobiology and Methodology of Behavioral Sciences, Faculty of Psychology, University of Malaga, Málaga, Spain; ^3^Department of Spanish Language I, University of Malaga, Málaga, Spain; ^4^Department of Neurology and Sant Pau Biomedical Research Institute, Hospital de la Santa Creu i Sant Pau, Universitat Autònoma de Barcelona, Barcelona, Spain; ^5^Center for Networked Biomedical Research into Neurodegenerative Diseases, Madrid, Spain; ^6^Neurophysiology Unit, Regional University Hospital Carlos Haya, Málaga, Spain

**Keywords:** dynamic aphasia, congenital mirror movements, developmental cerebral anomalies, neuroimaging, brain stimulation

## Abstract

The acquisition and evolution of speech production, discourse and communication can be negatively impacted by brain malformations. We describe, for the first time, a case of developmental dynamic dysphasia (DDD) in a right-handed adolescent boy (subject D) with cortical malformations involving language-eloquent regions (inferior frontal gyrus) in both the left and the right hemispheres. Language evaluation revealed a markedly reduced verbal output affecting phonemic and semantic fluency, phrase and sentence generation and verbal communication in everyday life. Auditory comprehension, repetition, naming, reading and spelling were relatively preserved, but executive function was impaired. Multimodal neuroimaging showed a malformed cerebral cortex with atypical configuration and placement of white matter tracts bilaterally and abnormal callosal fibers. Dichotic listening showed right hemisphere dominance for language, and functional magnetic resonance imaging (fMRI) additionally revealed dissociated hemispheric language representation with right frontal activation for phonology and bilateral dominance for semantic processing. Moreover, subject D also had congenital mirror movements (CMM), defined as involuntary movements of one side of the body that mirror intentional movements of the other side. Transcranial magnetic stimulation and fMRI during voluntary unimanual (left and right) hand movements showed bilateral motor cortex recruitment and tractography revealed a lack of decussation of bilateral corticospinal tracts. Genetic testing aimed to detect mutations that disrupt the development of commissural tracts correlating with CMM (e.g., Germline DCC mutations) was negative. Overall, our findings suggest that DDD in subject D resulted from the underdevelopment of the left inferior frontal gyrus with limited capacity for plastic reorganization by its homologous counterpart in the right hemisphere. Corpus callosum anomalies probably contributed to hinder interhemispheric connectivity necessary to compensate language and communication deficits after left frontal involvement.

## Introduction

Children and adults with language and literacy impairments (specific language impairment, dyslexia, and autism spectrum disorders) tend to have weaker cerebral lateralization than neurotypically developing individuals (de Guibert et al., 2011; Bishop, 2013; Ogawa et al., 2019). In addition, there are differences in the evolution of developmental and acquired disorders in children (Temple, 1997; Luyster et al., 2011). Perinatal language impairments and acquired childhood aphasias due to unilateral lesions of the dominant hemisphere rarely lead to pervasive deficits because efficient (adaptive) neural plasticity promotes recovery (Rauschecker et al., 2009; Yeatman and Feldman, 2013). By contrast, the presence of long-lasting deficits is the rule in specific language impairments and this has been related to bilateral brain abnormalities (Vargha-Khadem et al., 1998; Guerreiro et al., 2002; Rapin et al., 2003; Soriano-Mas et al., 2009). In this respect, there is evidence of how multiple brain systems may sustain the same function (e.g., degeneracy – Noppeney et al., 2004; Stefaniak et al., 2019), which may explain cases of resilience of language/cognitive functions to brain lesions. The idea of degeneracy exists both within subject, aiding to compensate the damage to a given network, and over subjects as in normal neurodevelopmental variation that can result, for instance, in differences in hemispheric lateralization (Biduła et al., 2017). However, the existence of multiple degenerate systems does not have to mean that such systems can become efficient, a situation that may be particularly true in cases of developmental malformations (i.e., Oberman et al., 2012; Zsoter et al., 2012; Mainberger et al., 2013). Specific language impairments are associated with reduced or reversed functional lateralization of language networks (see references in Luyster et al., 2011), suggesting that both cerebral hemispheres are engaged to compensate language deficits through adaptive neural plasticity. Thus, neural adaptation may be less efficient in cases of bilateral brain abnormalities and might represent an earlier neural marker for developmental language disorders by interfering with the continuous acquisition of skillful language functions (discourse, functional communication).

Many developmental language disorders are not associated with gross structural brain changes, but speech-language delay may also be associated to unilateral, bilateral or diffuse developmental cortical anomalies (e.g., perisylvian cortical dysplasia) (Graff-Radford et al., 1986; Guerreiro et al., 2000, 2002; Barkovich, 2010). There is an association between language delay and developmental abnormalities of the cortical mantle and white matter tracts (Andrade et al., 2015; Paldino et al., 2015, 2016). Nevertheless, the characterization of language delay and its relationship with gross developmental brain anomalies has not been clearly defined.

The syndrome of dynamic aphasia (DA) is a subtype of transcortical motor aphasia (TCMA) (Goldstein, 1917; Kleist, 1934; Luria, 1977; Berthier, 1999; Alexander, 2006) usually associated with acquired focal brain lesions (stroke, neoplasms) (Robinson et al., 1998) or slowly progressive degenerative disorders (e.g., primary progressive aphasia) (Esmonde et al., 1996; Robinson et al., 2006) involving the left frontal lobe, basal ganglia, or both. In the original formulation of DA, Kleist (1934) described it as a syndrome characterized by reduced drive to generate propositional speech despite the relative preservation of other language functions including spontaneous speech, object naming, word and sentence repetition, comprehension, and oral reading (Luria, 1966, 1970). Luria segregated DA into different subtypes, but he did not delineate the differences from one another (Lebrun, 1995). It was Lebrun (1995) who separated DA into three subtypes; one subtype corresponded to typical TCMA, another subtype resulted from what Luria called “spreading activation syndrome” (i.e., an impaired selection between competing verbal items that hampers verbal production), and the last type was described as a lack of drive to generate language.

In the present case study, we focus on the last type of DA referred to as “lack of drive to generate language.” While all reported cases of DA were *acquired* (ADA) after brain injury or neurodegenerative disorders in adulthood (Alexander, 2006; Magdalinou et al., 2018), the case described herein resulted from developmental aberrations in both hemispheres mostly involving language-eloquent cortical regions and white matter tracts in a teenager male (subject D). Similar to other children and adolescents with developmental language disorders associated to bilateral cortical anomalies (Guerreiro et al., 2000, 2002), subject D was brought to our Unit by his mother complaining limited communicative ability. She claimed that “he does not speak spontaneously and is not communicative.” This case can be endorsed to the category of Developmental Language Disorder (DLD) (American Psychiatric Association, 2013; Bishop et al., 2017). It was noticeable, however, that the language disturbance in subject D did not fulfill the criteria for any type of DLD reported up to now. Since it rather seems to be similar to one of the three variants described in ADA (lack of drive to generate language) (Kleist, 1934; Alexander, 2006), after performing a comprehensive testing, we classified the language disorder in subject D as *developmental dynamic dysphasia* (DDD). In this boy, DDD co-occurred with other neurodevelopmental disorders (mild left hemiparesis and congenital mirror movements - CMM) (Méneret et al., 2014) which in our view does not invalidate the diagnosis of DDD (see Bishop, 2017). In fact, the primary complain was language delay and subject D had normal hearing by audiometry and an intellectual quotient (IQ) > 70 (see Guerreiro et al., 2002).

We performed a multimodal evaluation to identify the brain-cognitive profile of subject D including testing of cognitive, language, and motor functions. Multimodal neuroimaging included structural magnetic resonance imaging (MRI; high-resolution T_1_-weighted image), functional MRI (fMRI) during four different tasks (phonemic fluency, semantic decision and left and right finger tapping) that allowed to evaluate functional cerebral dominance for language and motor functions, and diffusion tensor imaging (DTI)-Tractography of white matter tracts, that enabled the visualization of the language and motor pathways. In addition, transcranial magnetic stimulation (TMS) and genetic testing were performed to detect mutations that disrupt the development of commissural tracts (e.g., Germline DCC mutations).

## Materials and Methods

### Case Description

Subject D was a 12-year-old right-handed boy with concurrent DDD and CCM who was brought by his mother to our Unit for language testing. She reported that subject D had “problems to verbally explain things… showing poor communication and sometimes making nonsense comments.” She provided information about family history and her son’s developmental milestones. The father of subject D was described as “shy and non-communicative.” The parents and the brother of subject D were also right handed. Subject D was the second born of non-consanguineous parents. He was the product of a full-term pregnancy of 38 weeks. Maternal age at delivery was 30 years old. Delivery was normal and subject D’s Apgar scores at 1 and 5 minutes after birth were 9 and 10, respectively. His birth weight was 3.500 g. Shortly after birth subject D developed a short-lived bilateral arm tremor that disappeared before hospital discharge 24 h later. Developmental milestones were slightly delayed for language, communication and motor functions.

During infancy, subject D was discovered to have several medical, neurological, ophthalmological and skeletal abnormalities. At 9 months of age he was operated on for bilateral inguinal hernia, and at 3 years-old he was operated of bilateral strabismus. CMM were discovered at age 4 in kindergarten. Skeletal and neurological exams at the ages of 8 and 12 years disclosed mild dorsal scoliosis, pectum carinatum and turricephaly. He also had mild developmental delay, mild left-sided hemiparesis, increased blinking and CMM of the opposite hand and foot during voluntary movements. Cognitive testing at school when subject D was 8.10 years, showed a verbal IQ of 73, below average performance in the Colored Raven Progressive Matrices (Raven et al., 1975) and limited vocabulary with impaired ability to define words. Subject D was right handed (+100) as assessed by the Edinburgh Inventory of Handedness (Oldfield, 1971).

The study was performed in compliance with the Declaration of Helsinki. The parents of subject D signed a written informed consent for participation in the study and for the publication of the results. The protocol of this study was approved by the Ethical Research Committee Provincial of Malaga, Spain.

### Cognitive and Intelligence Testing

Although subjects with *pure* cases of ADA with deficits confined to speech production have been described (Costello and Warrington, 1989; Gold et al., 1997; Robinson et al., 1998), others have more widespread speech and language deficits involving phonological, lexical and syntactical functions (mixed ADA) (Esmonde et al., 1996; Snowden et al., 1996; Raymer et al., 2002; Warren et al., 2003) and still others present with additional non-language cognitive deficits (Robinson et al., 2006; Caine et al., 2018; Magdalinou et al., 2018). Thus, the cognitive profile of DDD in subject D was also explored with tests tapping intelligence, concept formation and reasoning, memory, and executive functions.

#### Methods

Subject D was evaluated with the Wechsler Intelligence Scale for Children (WISC) (Wechsler, 1974) and the Raven Colored Progressive Matrices (RCPM) (Raven et al., 1975). Memory was examined with the Test of Memory and Learning (TOMAL) (Reynolds and Bigler, 1994) and executive functions were tested with the Trail-Making Test (TMT) (Reitan, 1958; Arango-Lasprilla et al., 2017), the Hayling Sentence Completion Test (HSCT) (Burgess and Shallice, 1997; Abusamra et al., 2007; Cartoceti et al., 2008), the Wisconsin Card Sorting Test (WCST) (Grant and Berg, 1948; Heaton et al., 2009) and the Stroop Test (Stroop, 1935).

#### Results

Table 1 shows the results of the cognitive evaluation. On the WISC, subject D performed in the inferior range in all three IQ scores and his performance was also impaired on the RCPM. Subject D’s learning and memory functions were also impaired with slightly lower scores in the verbal memory index than in the non-verbal memory index. On all tests tapping executive function, subject D had impaired performance. Analysis of the pattern of performance of subject D on the HSCT provided information on the mechanism underlying DDD. He was impaired in the two sections of the sentence completion task (HSCT) exclusively due to omissions and prolonged response times (>20 *s*). While he could successfully complete many open-ended sentences (0.73) in the sensible completion Section 1, he was totally unable to choose a word unrelated to both the syntactic and semantic context of the frame sentence in the unrelated completion Section 2, producing no responses to any sentence.

**TABLE 1:** Cognitive testing.

Tests	Subject *D*’s scores	Performance descriptor	Normative data
**Intelligence**			
Verbal IQ	77	Inferior	
Performance IQ	76	Inferior	
Full Scale IQ	78	Inferior	>5th%ile
Raven Colored Progressive Matrices (max: 36)	26	BA	25th%ile
**Memory**			
**Test of Memory and Learning (TOMAL)**			
Verbal memory index	73	BA	100 ± 15
Non-verbal memory index	81	BA	100 ± 15
Composite memory index	74	BA	100 ± 15
**Executive function**			
**Trail-Making Test**			
Part A (sec/errors)	58/1	BA	<5th%ile
Part B (sec/errors)	109/1	BA	<5th%ile
**Hayling Sentence Completion Test ^α^**			
Section 1 – sensible completion (max:	11/4*	A	–
15)/errors	0/15	BA	–
Section 2 – unrelated completion/errors			
**Wisconsin Card Sorting Test (64 cards)**			
Categories	3	BA	>16th%ile
Correct responses	46	–	
Perseverations	18	SBA	45th%ile
**Stroop Test**			
Word reading (score/errors)	46/0	BA	<25th%ile
Naming colors (score/errors)	38/1	BA	25th%ile
Word-Color	18	BA	
Interference	−2.81	BA	

^α^The Argentinian version by Abusamra et al. (2007) was used. *All errors were omissions in both Sections. Data from healthy children show that errors in Section 1 are around 1.9% and responses in Section 2 are 58% correct (Cartoceti et al., 2008).

### Orientation, Perception and Motor Tests

#### Methods

Several tests were administered to evaluate these skills. These included the Right-Left Orientation (RLO), Tactile Form Perception (TFP), Finger Localization (FL), and Judgment of Line Orientation (JLO) (Benton et al., 1983).

#### Results

On the RLO, he showed a flawless performance when he was asked to orient in his own body (12/12) but showed a severe confronting person defect (1/8). In the TFP, he had normal performance with the preferred right hand (9/10) and mildly impaired tactile perception with the non-preferred left hand (7/10) (Spreen and Gaddes, 1969). On the FL, he demonstrated no deficit in the identification of single fingers both with hidden hands (20/20) and with visible hands (19/20), but a mildly impaired performance on identifying two simultaneously touched fingers when the hand was hidden (11/20), particularly for the left hand (4/10). Overall, the total score is mildly impaired in this task (total: 50/60; age-matched controls from Wake, 1957: mean = 54.4; *n* = 70). Subject D had average performance on the JLO test (21/30; age-matched controls: 24.7 ± 3.8, Benton et al., 1983).

### Language Testing

#### Auditory Processing, Word Semantics, Receptive Vocabulary, Reading and Spelling

##### Methods

Auditory processing and word semantics were assessed with several subtests of the Spanish version (EPLA) of the Psycholinguistic Assessments of Language Processing for Aphasia (PALPA) (Kay et al., 1992; Valle and Cuetos, 1995). These included Non-word Minimal Pairs (PALPA 1), Word Minimal Pairs (PALPA 2), Repetition: Syllable Length (PALPA 7), Repetition: Non-words (PALPA 8), Repetition: Imageability × Frequency (PALPA 9), Sentence Repetition (PALPA 12), Digit Production/Matching span (PALPA 13) and Spoken Word-Picture Matching (PALPA 47). The receptive vocabulary ability was examined with the Peabody Picture Vocabulary-III (Dunn et al., 2010). Oral reading and spelling to dictation were tested also using PALPA subtests. Oral reading was tested for Letter Length (PALPA 29), Imageability × Frequency (PALPA 31), Grammatical Class (PALPA 32), and Grammatical Class × Imageability (PALPA 33), Morphological Endings (PALPA 34), Regularity (PALPA 35), and Non-words (PALPA 36). Spelling to dictation was tested for Letter Length (PALPA 39), Imageability × Frequency (PALPA 40), Morphological Endings (PALPA 43), Regularity (PALPA 44) and Non-words (PALPA 45). The PALPA has been originally designed for use with people with acquired disorders and hence it does not include developmental norms (Kay and Terry, 2004). One requisite for the diagnosis of ADA is that comprehension, naming and transcoding (repetition and oral reading) should be relatively spared or remarkably less impaired than spontaneous speech (Luria, 1966, 1977). Therefore, to be confident that performance on language domains in subject D was not so affected as spontaneous speech, subtests of the PALPA were compared with adult norms for Spanish speaking subjects (Valle and Cuetos, 1995). Results on these subtests were classified as “average” when scores were within 2 standard deviations or less from the mean (scores between 0.96 and 1.0 relative to normative data); “slightly below average” (scores between 0.90 and 0.95 relative to normative data), and “below average” (scores < 0.90).^1^ Scores on the Peabody Picture Vocabulary-III were compared with age-matched normative data (Dunn et al., 2010).

##### Results

Table 2 shows the results on tasks tapping auditory processing, word semantics and receptive vocabulary. Most scores on PALPA subtests (18 out of 21) ranged from average (12/21) to slightly below average (6/21) and only a few scores were below average (3/21). Subject D performance on Non-word Minimal Pairs (PALPA 1) and Word Minimal Pairs were preserved obtaining better scores in the latter. Scores in Repetition: Syllable Length (PALPA 7) and Repetition: Non-words (PALPA 8) were flawless and almost intact in the Repetition: Imageability × Frequency (PALPA 9) subtest where subject D only performed 4 errors and all of them were lexicalizations (e.g., “cuabro”→ *cuadro* [painting]). Sentence Repetition was also preserved, but Digit Production/Matching Span (PALPA 13) was mildly reduced. Performance on the Spoken Word-Picture Matching (PALPA 47) and the Peabody Picture Vocabulary-III were preserved. Oral reading and spelling to dictation were preserved in most tasks (Table 3).

**TABLE 2:** Language testing.

Tests	Subject *D*’s scores (proportion)	Performance descriptor	Normative data^1^
**Auditory Processing: Comprehension Tests**			
**Non-word minimal pairs (PALPA 1)**			
Same (*n* = 28)	24 (0.86)	BA	27.45 ± 0.99
Different (*n* = 28)	25 (0.89)	A	27.09 ± 1.24
**Word minimal pairs (PALPA 2)**			
Same (*n* = 28)	25 (0.89)	A	27.54 ± 1.27
Different (*n* = 28)	26 (0.93)	SBA	27.68 ± 0.76
**Auditory Lexical Decision: Imag x Freq (PALPA 5)**			
High imageability-High frequency (*n* = 20)	20 (1.0)	A	20.00 ± 0.00
High imageability-Low frequency (*n* = 20)	19 (0.95)	SBA	20.00 ± 0.00
Low imageability-High frequency (*n* = 20)	19 (0.95)	SBA	19.95 ± 0.21
Low imageability-Low frequency (*n* = 20)	13 (0.65)	BA	19.41 ± 1.15
Non-words (*n* = 80)	73 (0.91)	SBA	78.18 ± 1.95
*Spoken Word-Picture Matching (n* = *40) (PALPA 47)*	39 (0.97)	A	39.45 ± 1.67
*Peabody Picture Vocabulary Test*	135	BA	30th%ile
**Auditory Processing: Repetition Tests**			
*Repetition: Syllable Length (PALPA 7) (n* = *24)*	24 (1.0)	A	23.8 ± 0.23
*Repetition: Non-words (PALPA 8) (n* = *24)*	24 (1.0)	A	22.9 ± 0.64
**Words, Imag x Freq (PALPA 9)**			
High imageability-High frequency (*n* = 20)	20 (1.0)	A	20.00 ± 0.00^1^
High imageability-Low frequency (*n* = 20)	20 (1.0)	A	19.82 ± 0.65
Low imageability-High frequency (n = 20)	20 (1.0)	A	19.68 ± 1.02
Low imageability-Low frequency (*n* = 20)	19 (0.95)	SBA	19.27 ± 1.93
*Non-words (n* = *80)*	76 (0.96)	A	77.68 ± 3.35
*Repetition: Sentences (PALPA 12) (n* = *36)*	34 (0.94)	A	–
*Digit Production (PALPA 13)*	4	SBA	5.91 ± 0.67
*Matching Span (PALPA 13)*	5	A	6.18 ± 1.34

*All test are from PALPA unless specified; ^1^Normative data from Valle and Cuetos (1995). The “performance descriptors” have been obtained by comparison with normative data as follows: A indicates *average*, SBA *slightly below average*, and BA *below average* (see further details in text).*

**TABLE 3:** Oral reading and spelling.

Tests	Subject *D*’s scores (proportion)	Performance descriptor	Normative data^1^
**Oral Reading**			
*Oral Reading: Letter Length (PALPA 29)*	24 (1.0)	A	23.95 ± 0.21
*Oral Reading: Imag x Freq (PALPA 31)*			
High imageability-High frequency (*n* = 20)	20 (1.0)	A	19.95 ± 0.21
High imageability-Low frequency (*n* = 20)	19 (0.95)	SBA	19.95 ± 0.21
Low imageability-High frequency (*n* = 20)	20 (1.0)	A	19.95 ± 0.29
Low imageability-Low frequency (*n* = 20)	20 (1.0)	A	19.68 ± 0.55
*Oral Reading: Grammatical Class (n* = *40) (PALPA 32)*			
Nouns (n = 20)	19 (0.95)	SBA	19.95 ± 0.21
Adjectives (n = 20)	20 (1.0)	A	19.86 ± 0.34
Verbs (n = 20)	20 (1.0)	A	19.95 ± 0.21
Functional Words (n = 20)	19 (0.95)	A	19.77 ± 0.42
*Oral Reading: Grammatical Class x Imag (n* = *40)* (PALPA 33)			
Nouns (*n* = 20)	19 (0.95)	SBA	19.91 ± 0.29
Functional Words (*n* = 20)	19 (0.95)	SBA	20.00 ± 0.00
*Oral Reading: Morphological Endings (PALPA 34)*			
Regular Words (*n* = 30)	27 (0.90)	A	29.54 ± 1.30
Irregular Words (*n* = 30)	14 (0.53)	SBA	26.36 ± 5.84
*Oral Reading: Non-words (PALPA 36) (n* = *24)*	22 (0.92)	A	23.22 ± 0,69
**Spelling**			
*Spelling to Dictation: Letter Length (PALPA 39) (n* = *24)*	24 (1.0)	A	23.8 ± 0.23
*Spelling to Dictation: Grammatical Class (PALPA 41)*			
*Nouns (n* = *5)*	5	A	4.68 ± 0.55
Adjectives (*n* = 5)	5	A	4.91 ± 0.29
Verbs (*n* = 5)	5	A	4.91 ± 0.29
Functional Words (*n* = 5)	4	A	4.77 ± 0.52
*Spelling to Dictation: Grammatical Class x Imag (PALPA 42)*	9 (0.90)	A	9.73 ± 0.67
Nouns (*n* = 10)	8 (0.80)	SBA	9.82 ± 0.49
Functional Words (*n* = 10)			
*Spelling to Dictation: Non-words (PALPA 45) (n* = *24)*	24 (1.0)	A	22.54 ± 0.76

*All test are from PALPA unless specified; ^1^Normative data from Valle and Cuetos (1995). The “performance descriptors” have been obtained by comparison with normative data as follows: A indicates *average*, SBA *slightly below average*, and BA *below average* (see further details in text).*

### Speech Production

#### Naming for Nouns and Verbs

##### Methods

Oral naming was assessed by using black and white pictures from standardized naming batteries. In particular, noun naming was assessed with the standardized set of 260 pictures of the Snodgrass and Vanderwart (1980) battery, whereas verb naming was tested with 100 items from the Action Naming Battery (Druks and Masterson, 2000).

##### Results

The performance of subject D in noun naming was mildly impaired (214/260 [0.82]) in part due to the inclusion of items not known by subject D (i.e., footballhelmet, sled, spinningwheel). Error analysis mostly disclosed semantic errors (e.g., “envelope”→ *message*) (28 [0.61]) and omissions (14[0.30]), whereas other errors were rarely seen. There were 2 phonological (0.04), 1 formal (0.2) and 1 visual (0.02) errors. His performance in verb naming was preserved (91/100 [0.91]). Error analysis disclosed the production of a noun instead of a verb (e.g., “surf”→ *boat*) (5), and omissions which were always benefited with phonemic cueing (4).

#### Verbal Fluency

##### Methods

Phonemic verbal fluency was assessed with the Controlled Oral Word Association Task (F.A.S.) (Borkowski et al., 1967), and semantic fluency was assessed with two categories of living things (animals and fruits) and two categories of artifacts (clothes and transport).

##### Results

The performance of subject D in phonemic fluency was very poor since he was only able to produce three words in 3 min. In semantic fluency, his performance was also impaired in the four categories (animals: 9; fruits: 7; clothes: 5; transport: 5).

#### Narrative Production and Communication in Activities of Daily Living

##### Methods

A sample of picture-generated narrative was used. Subject D was asked to generate a story that corresponds to a novel scene depicting a picnic day with many people along the riverside, enjoying a picnic and performing different activities. The *Picnic Scene* from the Western Aphasia Battery (Kertesz, 1982) was used. Subject D was encouraged to describe the elements depicted in the card (nouns) as well as indicate the actions that the persons were doing (action verbs) during a time limit of 5 minutes. He was also encouraged to describe the scenes using sentences. The description was audio-taped and transcribed. The speech sample was analyzed for percentage of correct information units (%CIU) defined as non-redundant content words that convey correct information about the stimulus (Nicholas and Brookshire, 1993; Marchina et al., 2011; Zipse et al., 2012), using the following formula: number of CIUs/number of words × 100. According to Nicholas and Brookshire (1993) to be classified as CIUs, words should not only be intelligible in context, but also be accurate, relevant and informative with respect to the stimulus. Meaningless utterances, perseverations, paraphasias and other inappropriate information (exclamations) were counted as words, but not classified as CIUs. The duration of the narrative, the total number of words, the number of words per minute and the pauses were counted. Pauses ≥ 3 s were considered abnormal.

##### Results

The description of the picture was extremely poor. It contained 31 words produced in 53 s. Although the examiner requested subject D to be more explicative in two occasions, he was unable to add further information. Since there were no linguistic errors in the narrative, the number of words and CIUs were the same (31). There were 4 pauses, two of which were long (6.47. and 8.28 s). Subject D produced the following description of the Picnic Scene: *“They are having a snack*… *(2.51 s), a man is speaking, a comet with a dog*… *(6.47 s) there is a man fishing*… *(8.28 s), there are two men on a boat*… *(1.51 s) and there is a child collecting water.”*^2^

To examine communication in daily life, the mother of subject D was interviewed using questions of a communication scale developed for adults with aphasia (Communicative Activity Log; Pulvermüller and Berthier, 2008). The mother reported that her son had marked impairment in frequency and quality of communication in questions evaluating making statements or reports about facts, write down short notes, communicate when relaxed or under stressful situations and communicating with foreigners.

### Dynamic Dysphasia Testing

To elicit the typical language features of DA, an adaptation of a series of experimental tests developed by Robinson and co-workers to assess ADA (Robinson et al., 1998) was used. The original English version of these experimental tests was slightly modified and adapted to be administered to Spanish speaking individuals (Berthier et al., in preparation). Since these tests are experimental, they were also administered to a group of 10 healthy control adolescent boys matched by age (age range: 10–14; mean age ± SD: 11.87 ± 1.12; Crawford *t*-test, two tailed: *t* = 0.111; *p* = 0.914), handedness (all right handers), and years of schooling (although subject D needed additional classes and training, he did not repeat any academic course). The scores obtained by Subject D in each of these tasks were compared to those obtained by the control group using a two-tailed Crawford’s modified *t*-tests. This test allows comparing outcomes from one or more individuals with results derived from small control samples (Crawford and Howell, 1998; Crawford and Garthwaite, 2002; Crawford et al., 2010). Performance on these tests in subject D and healthy controls was analyzed in terms of number of correct responses. The methodology and results of these tests are described below.

#### Test A

##### Generation of a single word to complete a sentence

###### Methods

Two sets of sentences were used. The first included 20 high-constraint sentences with not many usable referent words (e.g., “bicycles have two …”) and the second set was composed of 20 low-constraint sentences with numerous usable referent words (e.g., “It is good to be …”). One point per item was given if the generated word was appropriate. Sentences were presented in a random order. Results: Subject D completed 17 out of 20 high-constraint sentences correctly (0.85). By contrast, his performance in the low-constraint sentences was poor, completing 5 out of 20 sentences (0.25). Even though there was no time limit for completion of open-ended sentences, all errors were omissions. When asked for the high number of omissions, subject D replied, “I cannot find words” or “no words come to my head.” The total score of subject D was low (44/80), whereas the control group scores (78.1 ± 1.91) were significantly better (Crawford *t*-test, two-tailed: *t* = −17.02, *p* < 0.001).

#### Test B

##### Generation of a sentence from a single word

###### Methods

In this task, subject D and controls were asked to produce a whole sentence containing the word spoken by the examiner. Ten common nouns (e.g., “apple”) and 10 verbs (e.g., “sleep”) were randomly presented. Proper names were not used. Two points per item were given if the generated sentence was complete and grammatically correct and one point if the sentence was correct but not very informative. Results: Subject D produced 18 out of 20 phrases correctly (0.90) and his score was 36/40, whereas the performance of the control group was flawless (40 ± 0.0) (Crawford *t*-test, two tailed: *t* = −38.13, *p* < 0.001).

#### Test C

##### Generation of a sentence from a given sentence context

###### Methods

In this task, subject D was asked to generate a second sentence around the theme of the first. For example, the sentence “Carmen is always smiling” could be followed by the sentence “because she is always very happy.” Twenty sentences were presented and one point per item was given if the generated sentence was complete, grammatically correct and thematically related to the first stimulus sentence. Results: The performance of subject D in this task was impaired. He did not generate a novel sentence in 11/20 occasions (0.55). In the remaining sentences, he used some words of the target sentence in the response, usually repeating the verb verbatim or changing the verb tense, indicating echo-answer^3^. The performance in the control group was better than in subject D (25.6 ± 9.2) but the difference did not reach statistical significance (Crawford *t*-test, two-tailed: *t* = −0.78, *p* = 0.45).

#### Test D

##### Generation of a sentence from a single picture

###### Methods

Subject D and the control group were presented with 10 pictures of common objects (e.g., an iron or an umbrella) and asked to produce a whole sentence incorporating the noun of the picture. One point per item was given if the generated sentence was complete (not to simply name the item), grammatically correct and related to the presented picture. Results: Subject D had a moderately impaired performance in this task as he failed to generate a sentence in 3 out of 10 examples (bicycle, eyeglasses, and rabbit) (0.30). In the remaining items, although the generation of the sentences were correct, responses were very simple (e.g., example: iron; generated sentence: “The iron is used for ironing”). In addition, it was noticeable that the generation of correct sentences was preceded by prolonged latencies (ranging from 3.73 to 24.58 s) in four sentences. The performance in the control group was 10 ± 0.0 (Crawford *t*-test, two-tailed: *t* = −57.20, *p* < 0.001).

#### Test E

##### Sentence given a pictorial scene

###### Methods

Subject D and the control group were asked to produce a sentence to describe simple pictorial scenes selected from the Object and Action Naming Battery (Druks and Masterson, 2000). Twenty pictorial scenes (e.g., a boy playing basketball, a dancing couple) were used. Two points per item was given if the sentence generated was complete, grammatically correct and related to the presented scene. Results: Subject D obtained a score of 28/40 (0.70), whereas controls’ performance was flawless (40 ± 0.0) (Crawford *t*-test, two-tailed: *t* = −114.41, *p* < 0.001).

#### Test F

##### Generation of sentences from a pictorial scene. what might happen next?

###### Methods

Subject D and controls were presented with simple pictures selected from the Object and Action Naming Battery (Druks and Masterson, 2000) and asked to generate a sentence describing what might happen next. For instance, a picture showing a man bleeding after being bitten by a dog would be followed by the sentence “he went to the hospital.” Twenty pictorial scenes (e.g., a boat sinking, a person tying the laces of his trainers) were presented. Two points per item were given if the generated sentence was complete, grammatically correct and it was not a mere description of the scene, but a prediction of what would follow the corresponding situation. One point was given for an incomplete description. Results: The performance of subject D was significantly worse (5/40, [0.12]) than the one achieved by the control group (39.8 ± 0.42) (Crawford *t*-test, two-tailed: *t* = −79, *p* < 0.001). The qualitative analysis showed that subject D was unable to generate a description in 11 frame pictures (0.55). In the remaining 9 frame pictures there were 2 correct descriptions, 2 incomplete and 5 descriptions of the picture.

#### Test G

##### Story generation from a pictorial context

###### Methods

Subject D and the control group were presented with simple pictures and asked to generate a brief story describing what might happen. Ten pictorial contexts (e.g., a man watering the plants, a woman petting a cat) were presented. One point per item was given if the generated speech consisted of two or more related or connected complete and grammatically correct sentences. Results: As expected from the results obtained by subject D in Test F, he was totally unable to generate any story. Therefore, the test was interrupted after five consecutive failures. The performance of the control group was normal (20 ± 0.0), all of them generated brief meaningful and very illustrative stories.

### Neuroimaging

#### Functional Activations Related to Language and Motor Functions

##### Methods

(1) MRI data acquisition. MRI data was acquired on a on a 3-T MRI whole-body scanner (Philips 3T Intera, Release 3.2.3.1, with an eight-channel platform) equipped with a six-channel Philips SENSE head coil. Head movements were minimized using head pads and a forehead strap. First, high-resolution T1-weighted structural images of the whole brain were acquired with the following parameters: TR = 10.03 ms, TE = 4.606 ms, slice thickness 0.8 mm, 200 slices, voxel size: 0.75 × 0.75 × 0.8, flip angle = 8°, matrix size 320 × 320 × 200. Then, Diffusion Tensor Imaging (DTI) acquisition was performed using multi-slice single-shot spin-echo echo-planar imaging (EPI) with specific parameters as follows: matrix size 128 × 128 × 65, an acquisition voxel of 1.67 mm × 1.67 mm × 2.00 mm, TE = 91 ms, TR = 11621 ms, b factor = 800, flip angle 90°. After the acquisition of the structural data, four different fMRI were carried out following a block design. Each task involved a functional run consisting on 100 functional images (FFE/EPI sequence with epi factor 35, TR = 3000 ms and TE = 35 ms and flip angle 90°. The image matrix was 64/64 r. 30 axial slices were acquired for each volume, with a 4 mm slice-thickness and no gap. Voxel size was 1.8 mm x 1.8 mm x 4 mm). (2) fMRI experimental design. (2a) To evaluate the brain functional correlates of the language function in subject D, two different fMRI paradigms were used, one to study the functional correlates of phonological fluency and another one of semantic decision. Language production and comprehension may follow a different lateralization pattern (e.g., left hemisphere for production and comprehension, and right hemisphere for comprehension), as it has been shown in healthy subjects (Bernal and Ardila, 2009; Lidzba et al., 2011) and in individuals with developmental brain anomalies (see Berthier et al., 2011). For the Phonological fluency Task, subject D was required to mentally evoke as many words as possible beginning with a specific letter. At the beginning of each active block, the participant heard a letter, and then he was instructed to start producing the words. The letter was different for each block (F, A, S…). In the Semantic Decision Task, thirty draws of animals were presented (6 in each block) via MRI compatible goggles and the participant was required to move the right finger if he saw an animal that was a farm animal. Half of the animals were farm animals and the other half were not. (2b) In addition, a finger tapping paradigm was used to evaluate the CMM. Two fMRI runs, one for the left hand and another for the right hand, were carried out. The four functional runs were presented following identical fMRI block designs which consisted of five task blocks interspersed with five blocks of rest in which the participant was just required to stop doing the task and simply wait with the eyes open looking to a fixation point. The fMRI experiment followed a block design in order to measure the sustained brain responses related to the studied language and motor processes. Each block (task and rest blocks) lasted 30 s, therefore the total duration of each run was of 5 min. During the active block, the participant was required to perform a self-paced unimanual finger-tapping task. One run required finger tapping of the right hand (Right finger Tapping Task), and the other required the movement of the left-hand finger (Left Finger Tapping Task). (3) fMRI pre-processing and analysis. Functional imaging data were pre-processed using standard procedures implemented in the Statistical Parametric Mapping software (SPM12)^4^. The same processing steps were performed for each functional run corresponding with each task. Both the high-resolution structural T1 image, and the fMRI runs were AC-PC oriented. Functional images of each run were realigned to the first scan of each series. The functional scans were co-registered to the T1 image. T1 image was segmented into different tissues, and the parameters derived from the segmentation were used for the normalization of the T1-weighted and the functional images. Finally, all functional images were spatially smoothed with an 8-mm FWHM kernel. Then, two conditions were specified for each task/run: Task and Rest. The mean timelines of BOLD signal in white matter and cerebrospinal fluid were included in the model as covariates together with realignment parameters, to remove signal from non-neural sources. The general linear model was applied to find activations of interest using the contrast: Task > Rest. Unless otherwise stated, all statistical results are reported at *p* < 0.001 uncorrected for multiple comparisons at the whole-brain level, with a minimal cluster extent of 20 voxels.

##### Results

###### Morphological description of the brain

**FIGURE 1:**
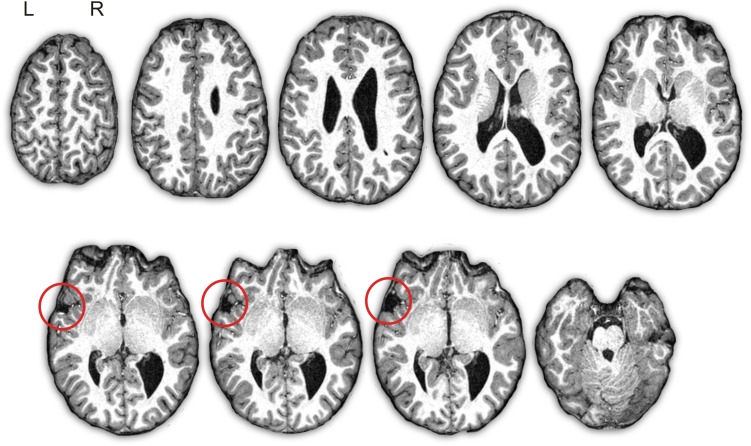
Depiction of subject D’s structural T1-weighted images. Axial slices of the brain in native space show the asymmetry of the volume of the lateral ventricles (right > left) with right occipital colpocephaly. There is an “open operculum sign” in the left hemisphere due to arrested development of the inferior frontal gyrus and superior temporal gyrus with exposure of the insular cortex (red circle). L, left; R, right.

A visual inspection of the T1-weighted anatomical image of subject D showed several developmental brain anomalies (Figure 1). The brain of subject D showed dilated right lateral ventricles, with hypertrophy of bilateral thalamus, caudate head and putamen. In addition, in the left hemisphere, he showed an open frontal operculum, the sylvian fissure was short and it ended in a marked ascending direction compared to a normal brain. Consequently, the left perisylvian area was reduced and the frontal gyri, the posterior temporal gyrus and the inferior parietal cortex were displaced. The right hemisphere seems to be larger than the left one and there was a right occipital petalia, findings already reported in children with specific language impairments (Soriano-Mas et al., 2009).

###### Brain activation during phonological fluency task

**FIGURE 2:**
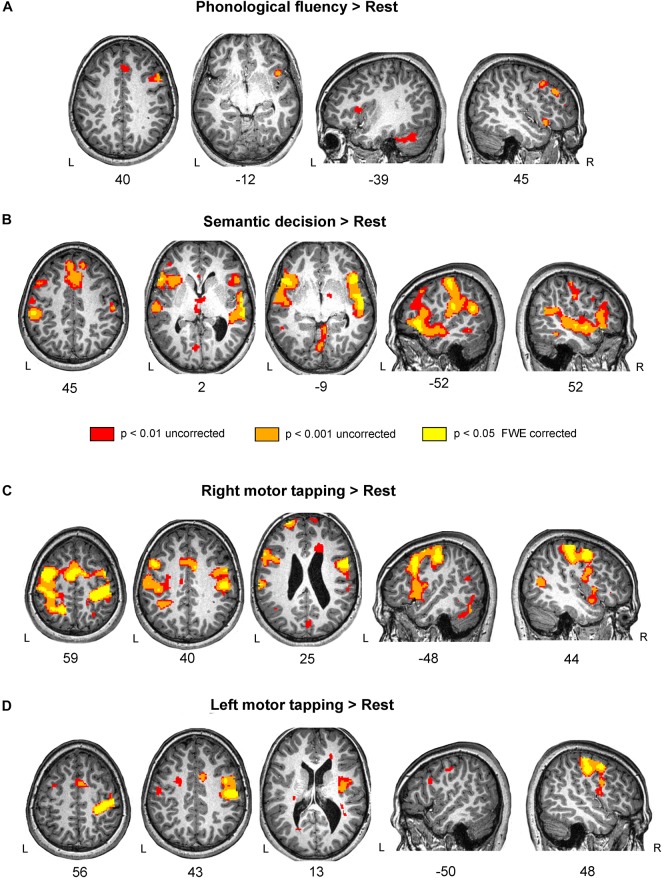
Brain activation during language and motor tasks in subject D. **(A)** Phonological fluency vs. Rest contrast showed a restricted pattern of activation, mainly in the right frontal lobe. Notice that subject D had a marked deficit in fluency tasks, which correspond with the weak activation pattern during the task. **(B)** Enhanced fMRI activity for the Semantic decision vs. Rest contrast was found in a bilateral network involving frontal, temporal and parietal areas. **(C)** Activation in the bilateral pre- and post- central gyri and Supplementary Motor Area (SMA) on the Right motor tapping vs. Rest contrast. **(D)** Left motor tapping vs. Rest contrast revealed increased activity in the right pre-and post-central gyri as well as in the SMA. Results are shown at three different thresholds: *p* < 0.05 corrected; *p* < 0.001 uncorrected; and *p* < 0.01 uncorrected threshold, with 20 voxels cluster extent. Results are shown in standard space over subject D’s normalized T1-weighted image. L, left; R, right.

The activation pattern associated to phonological fluency mainly involved areas of the right frontal lobe, such us the inferior and middle frontal gyri, and the left cerebellum (Figure 2A and Table 4). Two clusters of increased activation in left hemisphere appeared only with an uncorrected *p* < 0.01 threshold (Figure 2A). An overlap of the activation found in subject D in the phonological fluency task vs. rest contrast with a map resulting from a meta-analysis of fMRI studies focused on verbal fluency is reported in the Supplementary Figure S1A.

**TABLE 4:** Brain activations during the semantic decision, phonological fluency, left motor tapping and right motor tapping tasks.

Contrast	Cluster	Brain areas	Coordinates (Cluster peak)			Cluster size (no. of voxels)	FWE *p*-value (cluster level)	Unc. *p*-value (peak level)
			*x*	*y*	*z*			
**Phon. fluency vs. Rest**	1	R IFG pars triangularis	46	32	30	51	0.646	0.000
	2	L cerebellum Crus 1	−46	−60	−36	49	0.669	0.000
	3	R precental gyrus	28	41	20	48	0.681	0.000
	4	R IFG orbitalis	46	18	−14	37	0.808	0.000
	5	R middle frontal gyrus	30	44	22	45	0.716	0.000

	1	R IFG orbitalis, R superior temporal pole, R superior temporal gyrus	46	24	−14	2994	0.000	0.000
	2	L superior temporal gyrus, L inferior parietal cortex, L middle temporal gyrus	−58	−32	20	2553	0.000	0.000
	3	L superior frontal gyrus	−22	62	20	366	0.000	0.000
	4	L cerebellum (Crus 2 and 1), R cerebellum	−8	−88	−24	2943	0.000	0.000
	5	L IFG triangularis, L superior temporal gyrus	−54	24	−2	2131	0.000	0.000
	6	R SMA, L SMA	10	8	66	463	0.000	0.000
	7	L medial superior frontal gyrus	2	26	42	648	0.000	0.000
	8	R precentral gyrus	137	42	8	36	0.09	0.000
	9	Midbrain	−6	−30	−20	743	0.000	0.000
	10	R middle frontal gyrus,	34	42	24	162	0.054	0.000
**Semantic decision vs. Rest**	11	R inferior temporal gyrus (occipito-temporal)	50	−46	−20	27	0.97	0.000
	12	R posterior cingulate gyrus, precuneus	18	−44	32	398	0.001	0.000
	13	Cerebellum (vermis)	6	−52	−8	35	0.82	0.000
	14	Midbrain	10	−28	−16	73	0.40	0.000
		R superior frontal gyrus	20	62	28	20	0.95	0.000
	15	R inferior temporal gyrus (occipito-temporal)	−56	−52	−16	21	0.95	0.000
	16	R anterior parahippocampal gyrus	20	−10	−32	20	0.95	0.000
	17	R medial superior frontal gyrus	12	42	44	30	0.87	0.000
	18	R postcentral gyrus	56	−22	50	53	0.6	0.000
		L middle frontal gyrus	−54	18	40	27	0.9	0.000
	19	L anterior cingulum	0	38	22	23	0.93	0.000

**Right tapping vs. Rest**	1	R cerebellum, L cerebellum	14	−72	−44	338	0.002	0.000
	2	R superior frontal gyrus, R postcentral gyrus, R precentral gyrus	20	−10	72	5477	0.000	0.000
	3	L superior parietal cortex, L precentral gyrus,	−32	−52	64	6322	0.000	0.000
	4	R cerebellum, vermis, L cerebellum	22	−56	−26	2805	0.000	0.000
	5	L superior frontal gyrus	−26	62	22	192	0.025	0.000
	6	R posterior middle temporal gyrus	44	−66	12	87	0.278	0.000
	7	R insula, R rolandic operculum	48	10	0	122	0.121	0.000
	8	L lingual gyrus	−8	−92	−14	68	0.433	0.000
	9	L middle occipital gyrus	−42	−76	18	27	0.9	0.000
	10	L inferior temporal gyrus	−54	−54	−16	51	0.62	0.000
	11	L middle temporal gyrus	−42	−56	18	40	0.76	0.000
	12	R calcarine	8	−74	18	189	0.027	0.000
	13	L putamen	−18	−4	−10	36	0.8	0.000
	14	Midbrain	−4	−20	−22	162	0.049	0.000
	15	R lingual gyrus	20	−64	−27	74	0.37	0.000
	16	L cerebellum (Crus 1)	−48	−68	−26	22	0.94	0.000
	17	R insula	42	6	−18	41	0.74	0.000

**Left tapping vs.**	1	R superior frontal gyrus, R SMA	18	−8	74	432	0.000	0.000
**Rest**	2	R postcentral gyrus, R precentral gyrus	28	−32	52	2216	0.000	0.000
	3	L cerebellum	−12	−68	−44	27	0.9	0.000
	4	L cerebellum	−6	−48	−14	534	0.000	0.000
	5	R insula, R rolandic operculum	38	−8	12	145	0.06	0.000
	6	R cerebellum	20	−60	−24	104	0.16	0.000
	7	L postcentral gyrus	−40	−20	28	46	0.66	0.000

*All results are reported *p* < 0.001, uncorrected for multiple comparisons at the voxel level, with a minimum cluster extent of 20 voxels. Likewise, the results that are also corrected for multiple comparisons (FWE, *p* < 0.05) are also reported. Peak coordinates are reported in MNI coordinates.*

###### Brain activation during semantic decision task

Semantic decision activated a network comprising the typical ventral language stream bilaterally (see for instance Saur et al., 2008; López-Barroso et al., 2015) (Figure 2B and Table 4). These areas include bilateral IFG, both pars triangularis and pars opercularis, the anterior temporal lobe, the anterior and posterior superior temporal gyrus and the inferior parietal cortex. An overlap between the activation found in subject D in the semantic decision task vs. rest contrast and a map resulting from a meta-analysis of fMRI studies focused on semantics is reported in the Supplementary Figure S1B.

###### Brain activation during right motor finger tapping task

Subject D showed a bilateral pattern of activation involving the pre- and post- central gyri in both hemispheres as well as bilateral SMA, IFG and cerebellum. Results are reported in Table 4 and Figure 2C.

###### Brain activation during left motor finger tapping task

Subject D showed a robust activation in the right precentral and postcentral gyri, and small clusters of activation in the right insula and bilateral cerebellum. All significant results are reported in Table 4 and Figure 2D.

#### Functional Lateralization Indexes for Language and Motor Tasks

##### Methods

A lateralization index (LI) was calculated considering the activation difference between the left and right sides throughout different regions of interest (ROIs). ROIs were defined using WFU-Pickatlas toolbox^5^;Maldjian et al., 2003). For the four contrasts (Phonological fluency vs. rest, Semantic Decision vs. rest, Left Tapping vs. rest, and Right Tapping vs. rest), a LI was calculated using different ROIs: Hemisphere ROI (the whole right and left hemispheres) was used to calculate the LI in the four contrasts, IFG ROI (i.e., corresponding to Broca’s area) was used to explore the LI in the two language contrasts, and the precentral gyrus ROI (i.e., corresponding to primary motor cortex) was used to calculate the LI on the two motor contrasts. The formula used to calculate the LI was: (Right – Left)/(Right + Left)^∗^100, where Left and Right indicated the number of activated voxels in the corresponding left and right ROIs, respectively. The threshold used for the LI was identical as the one used for the contrasts (*p* < 0.001, uncorrected). The lateralization index ranges between -100 (extreme left lateralization) and 100 (extreme right lateralization). Values between −20 and 20 represent bilateral activation, and positive above 20 indicates left lateralization. This cut off to classify the patterns of lateralization was based on previous studies (Binder et al., 1996; Springer et al., 1999).

##### Results

**FIGURE 3:**
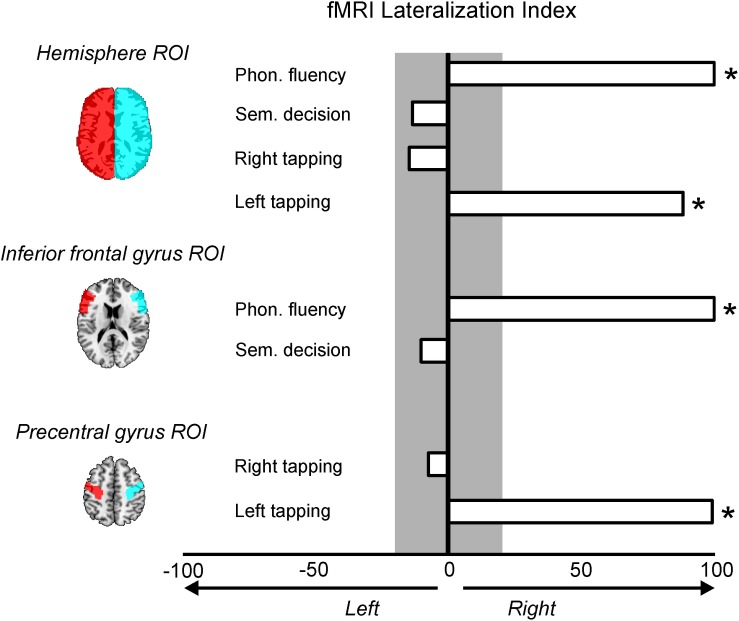
Lateralization indexes (LI) for the four fMRI contrasts and the region of interest (ROI) used. LI values greater than 20% mean right lateralization and are marked with an asterisk; LI values greater than –20% mean left lateralization and no LI was found with this pattern. Values between –20 and 20% (gray color) mean a symmetrical pattern of activation.

In subject D, the LI comparing Phonological Fluency vs. rest contrast disclosed that the LI was 100 for both analyses, using the Hemisphere and the IFG ROIs, thus showing an extreme right lateralization in both cases (Figure 3). The LI for the activation related to semantic decision revealed that for the hemisphere ROI, the LI was of −13%, and of −10% when using the IFG ROI. Therefore, subject D

showed a bilateral pattern of activation during semantic decision (Figure 3). The LI for the activation related to right motor tapping was −14% when analyses were restricted to the whole hemispheres, and −7% when they were restricted to the precentral gyrus ROIs. Both LIs suggest a symmetrical activation pattern (Figure 3) for the right motor tapping task. Finally, the LI for the activation related to left motor tapping was of 100% using both the hemisphere ROIs and the precentral gyrus ROIs, suggesting an extreme right lateralization (Figure 3).

#### Diffusion Tensor Imaging (DTI) Pre-processing

Diffusion data pre-processing started with motion and eddy current correction as using FMRIB’s Diffusion Toolbox (FDT) (Smith et al., 2004; Woolrich et al., 2009), and the Brain extraction was performed with the Brain Extraction Tool (BET), both parts of the FMRIB Software Library (FLS)^6^. Diffusion tensor estimation was carried out using Diffusion Toolkit’s least-square estimation algorithm for each voxel (Ruopeng Wang and Van J. Wedeen, TrackVis.org, Martinos Center for Biomedical Imaging, Massachusetts General Hospital). Whole-brain tractography used an angular threshold of 35° and an FA threshold of 0.15. A fractional anisotropy (FA) map was generated using Diffusion Toolkit.

##### Deterministic tractography

###### Methods

Different white matter tracts were selected as tracts of interest due to their implication in language or motor functions, and consequently they were reconstructed and examined. Specifically, as tracts related to language, we selected the three segments of the arcuate fasciculus (AF) (long, anterior, and posterior) and the frontal aslant tract (FAT) as dorsal language pathways; while the inferior fronto-occipital fasciculus (IFOF) and the uncinate fasciculus (UF) were selected as ventral language pathways. Referred to the motor function, we examined the corpus callosum and the corticospinal tracts. Virtual dissections of the tracts were performed using the software TrackVis^7^. Spheres a hand-drawn ROIs were defined over the FA or FA color maps and used to isolate single tracts following previously reported procedures (Catani and Thiebaut de Schotten, 2008; López-Barroso et al., 2013). When required, spurious fibers were removed from the main fiber tracts by using an additional avoidance ROI. All tracts were dissected in native space and in both cerebral hemispheres.

###### Results

**FIGURE 4:**
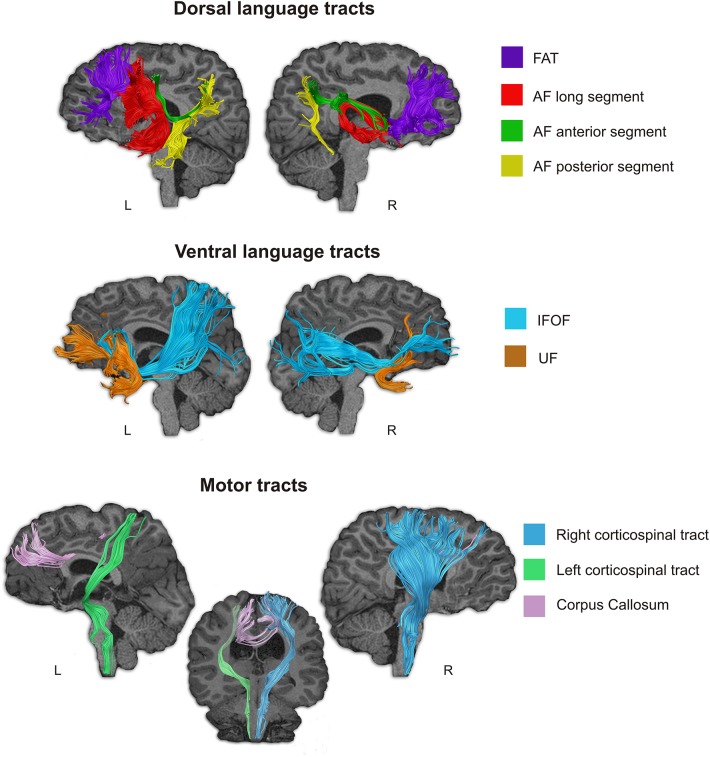
Language-related and motor-related white matter pathways of subject *D* were tracked using deterministic Tractography. Notice that all studied language related pathways (dorsal and ventral) could be reconstructed, however the three segments of the AF showed an atypical distribution. Although they connect frontal, parietal and temporal regions, since these areas were displaced in Subject *D* due to the morphological abnormality in his brain, the shape of these tracts is abnormal. The motor tracts were also reconstructed, but we could not find the pyramid decussation normally found in the caudal part of the medulla oblongata.

All the tracts were intact (Figure 4) and could be virtually reconstructed contrary to what happens when there is a brain injury, however, the morphology of some of these tracts was atypical. In the left hemisphere, among the dorsal tracts the FAT was reconstructed and it showed a normal morphology; the long, the anterior and the posterior segments of the AF were voluminous, especially the long AF segment, but accordingly to the shape of the sylvian fissure in the left hemisphere, both the frontal and the temporal branches were shorter than in a normal brain, while the dorsal terminations of the frontal branches of the anterior and the long segments reached the superior frontal gyrus. The posterior and anterior segments terminated in the inferior parietal cortex, but their atypical shape was a consequence of the displacement of these cortical areas (Figure 4). In the right hemisphere, the FAT showed a typical shape, whereas again the three segments of the AF showed an atypical pattern, associated to the atypical morphology of the perysilvian cortex. The ventral tracts were reconstructed in both hemispheres (Figure 4). The UF and IFOF of the left hemisphere showed greater volume than in the right hemisphere, following the pattern found for the AF.

The studied motor tracts were also successfully reconstructed (Figure 4). With the current methodological resolution, we were not able to find evidence for pyramid decussation at the level of the medulla oblongata. The right corticospinal tract (CST) showed greater volume than the left CST. The corpus callosum that connect the motor areas from both hemispheres was displaced, thus we could not reconstruct direct connections between contralateral frontal motor areas.

##### Dichotic listening

###### Methods

Dichotic listening was evaluated with a Spanish version of the three-pair dichotic listening task (DLT) (Strouse and Wilson, 1999; Zenker et al., 2007). Before performing this task, subject D underwent a tonal audiometry which revealed normal hearing bilaterally. Subject D was presented with a series of one to three pairs of numbers. Each pair consists of one number (from one to ten) presented on the left ear and a different number (from one to ten) presented on the right ear. After each number was presented, subject D was required to orally repeat which digits he has heard in each ear. Based on the DLT, Zenker et al. (2007) obtained the LI, which is computed as: LI = [(Right-Left)/(Right + Left)]^∗^100, where Right and Left are computed as the total number of individual digits recognized presented respectively to the right and left ears. In Zenker et al.’s (2007) study, the LI was 17% for the 6–12 age group and 5% for the 13–19 age group.

###### Results

In the DLT, subject D recognized 30% of the digits presented to the left ear, and only 19% of the digits presented to the right ear. The LI was -55% (i.e., strongly right brain lateralized), clearly below the scores of his age group (i.e., 17%). This means that subject D was less able to detect stimuli processed in the left hemisphere than in the right hemisphere, thus suggesting that his right hemisphere was dominant for auditory processing. These results complemented the findings from fMRI, which showed right hemisphere lateralization for speech production.

#### Transcranial Magnetic Stimulation

##### Methods

Motor evoked potentials (MEP) to the four limbs were obtained simultaneously using a transcranial magnetic stimulation (TMS) with a monopulse stimulator (Magstim 100) with a round coil (12 cm). The coil was placed tangentially to the scalp with its center over the vertex for cortical stimulation, and spinal roots were stimulated at C6–C7 and L4–L5 spaces while recording at the same positions bilaterally over the target muscles (1st dorsal interosseous and tibialis anterior muscles) with surface electrodes. Central conduction time (CCT in milliseconds) was measured as the difference between total and peripheral motor conduction time. The amplitude (μv) of the cortical response was measured as the average at least 3 supramaximal responses and as an amplitude ratio with the compound motor action potential (CMAP) electrically elicited (ZAMPR). For identification of cortical silent periods (CSP) the same protocol as for eliciting the MEP (while subject D performed a maximal voluntary contraction) was used. The CSP was quantified as the time elapsed between the onset of the MEP and the time at which the post-stimulus background EMG returned to the pre-stimulus mean amplitude (Poston et al., 2012).

##### Results

The TMS disclosed cortical bilateral responses with the same latency and amplitude for both 1st dorsal interosseous muscles with unilateral stimulation of hand motor cortical area, with a normal threshold (greater when stimulating the right hemisphere). Stimulation of both hemispheres showed a markedly diminished cortical silent periods (CSP) for both muscles. Values of different parameters (e.g., motor threshold, central conduction time, MEP latency, MEP cortical amplitude) obtained for hands and feet of subject D are presented in Supplementary Tables S1–S4.

#### Genetic Testing

##### Methods

Genetic testing was performed to detect mutations that disrupt the development of commissural tracts (i.e., Germline DCC mutations) and are associated with CMM (Méneret et al., 2014). The coding and flanking intronic regions of DCC (deleted in colorectal carcinoma [OMIM ^∗^120470]), RAD51 (RAD51 recombinase [OMIM ^∗^179617]), and DNAL4 (Dynein Axonemal Light Chain 4 [OMIM ^∗^ 610565]), were amplified by polymerase chain reaction (PCR) and Sanger sequenced on an ABI 3100 automatic sequencer (Applied Biosystems, Foster City, CA, United States). Resulting electropherograms were visually analyzed using Sequencher software (Gene Codes Corp. Ann Arbor, MI, United States). Primer pair sequences and PCR conditions are available under request.

##### Results

The genetic study did not disclose any pathogenic variant in the three analyzed genes (DCC, RAD51, and DNAL4).

## Discussion

We described, for the first time, the case of an adolescent boy who met diagnostic criteria for DLD (DSM-V, code: 315.32 [F80.2]; American Psychiatric Association, 2013) showing a profile of language impairment resembling DA. In all previous reports of ADA it was associated with focal brain lesions (tumors, stroke) and cortical-subcortical atrophy secondary to progressive degenerative disorders mostly involving fronto-subcortical regions. However, in subject D the occurrence of such syndrome coexisted with a malformed brain. Therefore, the neurobiological underpinnings of DDD in this adolescent boy could be considered of developmental origin. In the next section we examine what could be the causal mechanisms that might underlie DDD.

### Mechanisms Underpinning Developmental Dynamic Dysphasia

Several competing theoretical interpretations have been advanced to account for ADA (see Robinson et al., 2006). Indeed, cases of ADA have variously linked to impaired verbal planning (Costello and Warrington, 1989; Bormann et al., 2008), impaired selection between competing verbal responses (Robinson et al., 1998, 2005), inadequate semantic strategy formation (Gold et al., 1997) and reduced spontaneous and intentional activation of lexical-semantic representations (Raymer et al., 2002; Cox and Heilman, 2011; Satoer et al., 2014). These disrupted mechanisms may explain the language-specific form of the syndrome (Robinson et al., 1998, 2015). Nevertheless, complementary proposals suggest that reduced speech production may also be related to domain-general deficits resulting from impairment in novel thought generation and deficits in fluent sequencing of novel thoughts (Robinson et al., 2006, 2015a,b; Bormann et al., 2008; Robinson, 2013). Interpretation of ADA within this broader framework coincide with the original formulation by Luria and Tsvetkova (1967), who viewed this syndrome as a condition derived from general executive and attentional impairments related to lesions in the frontal lobes. Therefore, it seems that some cases ADA may result from a hybrid mechanism that combines failures in domain-general and language-specific functions.

Another candidate mechanism to account for ADA is the impairment of automatic spreading activation of lexical items during production tasks. Luria introduced the term “spreading activation syndrome” for explaining a subtype of ADA (see Lebrun, 1995). In this context, the word “spreading” means that during speech production tasks (e.g., naming) many words are activated simultaneously interfering one each other while the subject is selecting which one should be produced (Dell, 1986; Thompson-Schill et al., 1997; Levelt et al., 1999; Moss et al., 2005; Silkes and Rogers, 2012; Anders et al., 2017; Schnur, 2017). In this connection, we recently studied an adult person with ADA due to a left opercular-insular infarction, who commented on that during language tasks “many words come to my mind, but I cannot decide which one to choose…” (Berthier et al., *in preparation*). However, this mechanism seems not to be the one that explain DDD in subject D, who instead reported that no words come to him during speech production tasks. Subject D was capable of carrying out most language tasks dependent upon external stimuli (naming, repetition), but creating and organizing a narrative was challenging for him. It seems that in the case of subject D a marked reduced activation of lexical items prevails as a possible explanation for the impaired ability to generate words and sentences in both real life and testing situations (see Alexander, 2006; Stuss and Alexander, 2007; Cox and Heilman, 2011; Silkes and Rogers, 2012). Moreover, cognitive testing in subject D revealed impaired performance in all tasks tapping executive functions (TMT, HSCT, WCST, ST) unveiling that dysfunctional domain-general mechanisms are also contributing to dDD.

The pattern of performance exhibited by subject D on the two-part sentence completion task (HSCT) and on experimental tasks for DA would further illuminate the putative mechanism of reduced activation of lexical items underlying DDD. The HSCT is thought to assess both initiation speed and response suppression (Burgess and Shallice, 1997); therefore, delays in completing the missing word (Section 1) and failures to inhibit a strongly activated response before generating a new unconnected one (Section 2) are the expected outcomes in persons with frontal lobe involvement (Robinson et al., 2015a, 2016). Analysis of this task in subject D revealed impaired performance in the two sections and errors were omissions. No automatic completions were produced in Section 2 and, instead, subject D produced no responses. Failure to generate a completion word has been associated with left frontal lesions (Robinson et al., 2015b, 2016) and represents a typical pattern of performance in individuals with ADA (Robinson et al., 2005). In the same vein, he performed significantly worse than healthy controls in experimental tests of DA (Robinson et al., 1998), particularly in the more demanding ones. One constant characteristic of subject D while performing these tasks was that he frequently remained silent when asked to produce a sentence or to generate a brief story. When he was asked why he did not produce the requested information, he said “I have no words…. Words don’t come to me.” Moreover, his performance on the picture-generated narrative and phonological and semantic fluency tasks were also extremely poor. Nevertheless, other language functions (i.e., semantic comprehension, repetition of words, non-words and sentences, noun and verb naming, oral reading and spelling) were slightly below average or average. This dissociation, characteristic of ADA (Robinson et al., 1998, 2006; Berthier, 1999), may also characterize DDD. Defective semantic strategy formation has been considered implicated in some case of ADA (Gold et al., 1997), but subject D was fully capable of activating semantic knowledge when given an external stimulus as demonstrated by his preserved ability to name nouns and verbs. This pattern of performance (failure in initiating and sustaining a response in the absence of external cues) in subject D may be indicative of failure to spontaneously active lexical semantic representations (Cox and Heilman, 2011) perhaps due to impaired attentional processes (energization) (Stuss and Alexander, 2007; Stuss, 2011; Barker et al., 2018).

### Pitfalls of Establishing Brain-Behavior Relationships in a Malformed Brain

The syndrome of ADA is uncommon (Robinson et al., 1998; Berthier, 1999; Alexander, 2006) and we envisage that a DDD, as the one found in subject D, may be even rarer because it coexisted with bilateral brain malformations that distorted the architecture and connectivity of networks mediating expressive language and communication. Nevertheless, piecemeal analysis of the different malformations may illuminate the mechanisms underlying DDD in the present case. In first place, we analyze the role of gyral abnormalities in the left operculum on speech production deficits. The structural MRI showed a short sylvian fissure with arrested development of the left fronto-temporal operculum and exposure of a hypoplastic insular cortex (open operculum) indicative of a cortical dysplasia (Tatum et al., 1989; Piven et al., 1990; Van Bogaert et al., 1998). Detailed visual analysis of thin slices in high-resolution MRI also revealed that the configuration of the right Sylvian fissure was also atypical. Functional imaging also showed atypical results. While healthy subjects activate the left IFG in fluency tasks, as revealed by the Neurosynth meta-analysis for the term “verbal fluency” (Supplementary Figure S1A), the fMRI acquired during a phonological fluency task in subject D revealed that increased activation in the left inferior frontal gyrus resulted only when using an uncorrected *p* < 0.01 statistical threshold during this language task compared to rest. Small foci of activation were found in the homologous contralateral gyrus at a lower statistical threshold *p* < 0.001 uncorrected). Although the fMRI experiment and the structure of the fluency task applied in this study did not allow to separate right from bad trials (i.e., sustained brain response was measured during the whole block in which the subject was instructed to mentally evoke as many words as possible), the atypical pattern of functional activation together with the fact that subject D showed a poor performance in fluency task, suggest that contralateral functional plasticity in this case has been maladaptive. These results suggest that in the presence of a dysfunctional left frontal cortex, the right anterior perisylvian area was not fully competent to subserve efficient communication. Thus, it seems that this cross-hemispheric plasticity (left → right) could compensate basic language operations (i.e., object and verb naming, repetition), but was not sufficient to guarantee more elaborated language and communication skills required for the generation of fluent discourse.

Early left hemisphere injury may result in functional reorganization that, although permits sparing of language and motor skills, may distort the development of right hemisphere functions (Sandson et al., 1994; Satz et al., 1994). Moreover, individuals with unilateral, bilateral or diffuse gyral abnormalities in the frontotemporal operculum, like the ones found in subject D, have language delay (Guerreiro et al., 2002) which may persist into adulthood (Graff-Radford et al., 1986; Guerreiro et al., 2000). In such cases, positron emission tomography shows altered (decreased, increased or both) metabolic activity in both cerebral hemispheres (Van Bogaert et al., 1998; Luat et al., 2006). In the same line, children with specific language impairments (developmental dysphasia) show lack of fMRI activation during category fluency, responsive naming and picture naming tasks in left perisylvian language areas with hyperactivation in the right inferior frontal gyrus, insula and caudate nucleus (de Guibert et al., 2011). Note that the compensation of language deficits by the right hemisphere in left brain-damaged children is variable and depends on the residual capacity of the left hemisphere to maintain some language function (see references in Reilly et al., 2013). Nevertheless, another influential factor for the expected bias of transferring language functions to the right hemisphere in cases with left hemisphere damage (developmental or acquired) would be the functional status of the right hemisphere. We suggest that DDD in subject D may have resulted from the left perisylvian dysgenesis and also for the limited capacity of the unfit right hemisphere to ensure the development and evolution of more elaborated aspects of oral expression (i.e., conversation, narrative discourse) (Berthier et al., 2012; Catani and Bambini, 2014; Lomlomdjian et al., 2017). In other words, impaired language generation (verbal adynamia) in subject D may have resulted from inefficient neural plasticity in both hemispheres. By contrast, auditory comprehension in subject D ranged from preserved to mildly impaired performance in most tasks and the fMRI showed that activation during a semantic decision task occurred in canonical areas mostly linked by the ventral stream (Saur et al., 2008; López-Barroso et al., 2015). These areas include bilateral IFG, both pars triangularis and opercularis, the anterior temporal lobe, the anterior and posterior superior temporal gyrus and the inferior parietal cortex. Supplementary Results revealed that although there was a substantial overlap between the meta-analysis fMRI results in healthy subjects for the term “semantic” and the results from the contrast Semantic Decision vs. rest in subject D (Supplementary Figure S1B), the pattern found in subject D was more bilateral. This higher overlap compared to the one observed for the fluency task is in line with the fact that subject D’s performance in comprehension and semantic tasks was acceptable, and in addition it would show some evidence that at least in some functions, the atypical brain configuration observed in this case can be functional. Nevertheless, since results from the supplementary Neurosynth fMRI meta-analysis come from heterogeneous studies (e.g., different population, different tasks), these results should be taken cautiously and interpreted as a whole with the rest of the image results and clinical characteristics reported.

In second place, we examine the putative role of the dysmorphic white matter tracts in DDD. Previous studies have shown a strong relationship between the failure to identify the left AF and language dysfunction in cases with developmental cortical gyral abnormalities (Andrade et al., 2015; Paldino et al., 2015, 2016). Poor development of the left FAT has been related to profound expressive language impairmentin a child with a sex-linked chromosomopathy (karyotype 49, XXXXY) syndrome (Dhakar et al., 2016) and therapeutic interventions improving speech production and everyday verbal communication in post-stroke aphasia correlated with structural plasticity of the FAT and direct segment of the AF (Berthier et al., 2017). While there is marked individual variability in the configuration of left and right white matter tracts in healthy subjects (Gharabaghi et al., 2009; Berthier et al., 2012), the spatial arrangement of most white matter bundles in subject D was atypical. We could retrieve all long-distance white matter tracts in both cerebral hemispheres, but their configuration was distorted adopting an architecture that markedly deviated from the normal pattern. The cumbersome arrangements of most retrieved white matter tracts in both cerebral hemispheres were probably the result of the non-canonical configuration of the cortical mantle. DTI-tractography also disclosed abnormal decussation of the CSTs, a finding that correlated in subject D with the CMM.

The study of neurophysiological correlates of CMM with TMS study showed that unilateral stimulation of hand motor cortical area disclosed cortical bilateral responses. There are some limitations of our TMS study. First, TMS was not guided by a neuronavigation software module. Therefore, we cannot confidently determine the position of the current with respect to the brain sulcus and surface of subject D. Second, the use of a round coil in our TMS study cannot rule out the simultaneous stimulation of the motor cortex in both hemispheres because of the coil structure. Third, we also found that stimulation of both cerebral hemispheres showed diminished cortical silent periods (CSP) for both muscles. CSP are indexes of corticospinal inhibition during a tonic muscular contraction probably representing a GABAb-mediated inhibitory neurotransmission (Tergau et al., 1999). Therefore, shortened CSP after unilateral TMS in subject D may reflect the output from the non-stimulated M1 so that both the activity of motor cortices (M1) was released with intended uni-manual movements (Cincotta et al., 2002). These bilateral responses were absent when studying the cortical stimulations for leg muscles. Genetic testing to identify mutations associated to altered development of commissural tracts (i.e., Germline DCC mutations) (Méneret et al., 2014) were negative.

### Is the Diagnosis of Developmental Dynamic Dysphasia Reliable?

There is a caveat about reliability of diagnosis when one describes a well-known yet rare syndrome occurring for the first time in association with a new pathological condition (abnormal brain development). Analysis of more cases is clearly needed to confirm or reject the accuracy of the diagnosis. Note, however, that original cases of ADA were invariably associated to focal lesions (tumors, stroke, trauma) (i.e., Costello and Warrington, 1989; Robinson et al., 1998) but several years later similar cases have been related to different neurodegenerative conditions (i.e., Parkinson’s dementia, progressive supranuclear palsy, corticobasal degeneration, prion diseases) (Kartsounis et al., 1991; Esmonde et al., 1996; Warren et al., 2003; Caine et al., 2018; Magdalinou et al., 2018). The syndrome of ADA is a variant of TCMA (Berthier, 1999) and cases of this syndrome of developmental origin have not been reported so far. However, developmental conduction aphasia has recently been reported in a group study showing that specific and long-lasting problems with speech repetition were similar to the syndrome reported in adults (Northam et al., 2018). Similarly, the foreign accent syndrome previously described associated to focal lesions (Moreno-Torres et al., 2016) and neurodegenerative disorders (Luzzi et al., 2008) has recently been reported as a developmental disorder (Mariën et al., 2009; Berthier et al., 2016; Keulen et al., 2016). The second point is that subject D had non-language cognitive and motor disorders that may cast doubts on the reliability of the diagnosis of DDD. The contribution of impaired performance on tasks tapping executive functions to DDD is in agreement with reports of ADA, which attributed such deficits to impaired domain-general mechanisms (Robinson et al., 2006). Nevertheless, we consider the presence of CMM and low intellectual function unrelated to DDD. In support of this argument, most cases of CMM are discrete and not disabling coursing without concomitant cognitive deficits (Méneret et al., 2014, 2015) and low IQ is a constant feature of patients with ADA of different etiologies (Robinson et al., 1998, 2006, 2015).

## Data Availability Statement

The raw data supporting the conclusions of this manuscript will be made available by the authors, without undue reservation, to any qualified researcher.

## Ethics Statement

The studies involving human participants were reviewed and approved by the Ethical Research Committee Provincial of Malaga, Spain. The participants provided their written informed consent to participate in the study.

## Author Contributions

All authors listed have made a substantial, direct and intellectual contribution to the work, and approved it for publication. MB, GD, MT-P, and DL-B were involved in conception and design, acquisition of data, or analysis and interpretation of data. LE, LM-C, DM-S, and PZ were involved in cognitive and language testing. JC and OD-I performed genetic testing. IM-T, VF, and MP performed the neurophysiological studies. MB and DL-B interpreted the neuroimaging data. MB, GD, and DL-B drafted the manuscript and revised it critically for important intellectual content.

## Conflict of Interest

The authors declare that the research was conducted in the absence of any commercial or financial relationships that could be construed as a potential conflict of interest.

## Footnotes

1 In the validation of the Spanish version of the PALPA, healthy subjects performed almost at ceiling. Thus, standard deviations were low indicating that the data points tended to be close to the mean and that failing only one item in a given test placed the scores of subject *D* two standard deviations below the mean.

2 This excerpt has been translated from Spanish. Note that the number of words has been counted in Spanish (*n* = 31), so that the number (*n* = 33) does not coincide with the English translation.

3 Echo-answer refers to the inadvertent repetition of words or sentence fragments of the target stimulus into the response, in general, with the purpose of improving auditory comprehension (Berthier et al., 2017). This was not the case in subject *D* because he had preserved auditory comprehension. It is possible that in this subject, echo-answer resulted from limited linguistic resources to generate novel sentences.

4 http://www.fil.ion.ucl.ac.uk/spm/

5 http://www.nitrc.org/projects/wfu_pickatlas/

6 www.fmrib.ox.ac.uk/fsl/

7 www.trackvis.org
