# Supplementary figures and images for: Developmental Dynamic Dysphasia: Are Bilateral Brain Abnormalities a Signature of Inefficient Neural Plasticity?
Source: Front Hum Neurosci. 2020 Mar 24;14:73. doi: 10.3389/fnhum.2020.00073 (PMC7107010; doi:10.3389/fnhum.2020.00073)

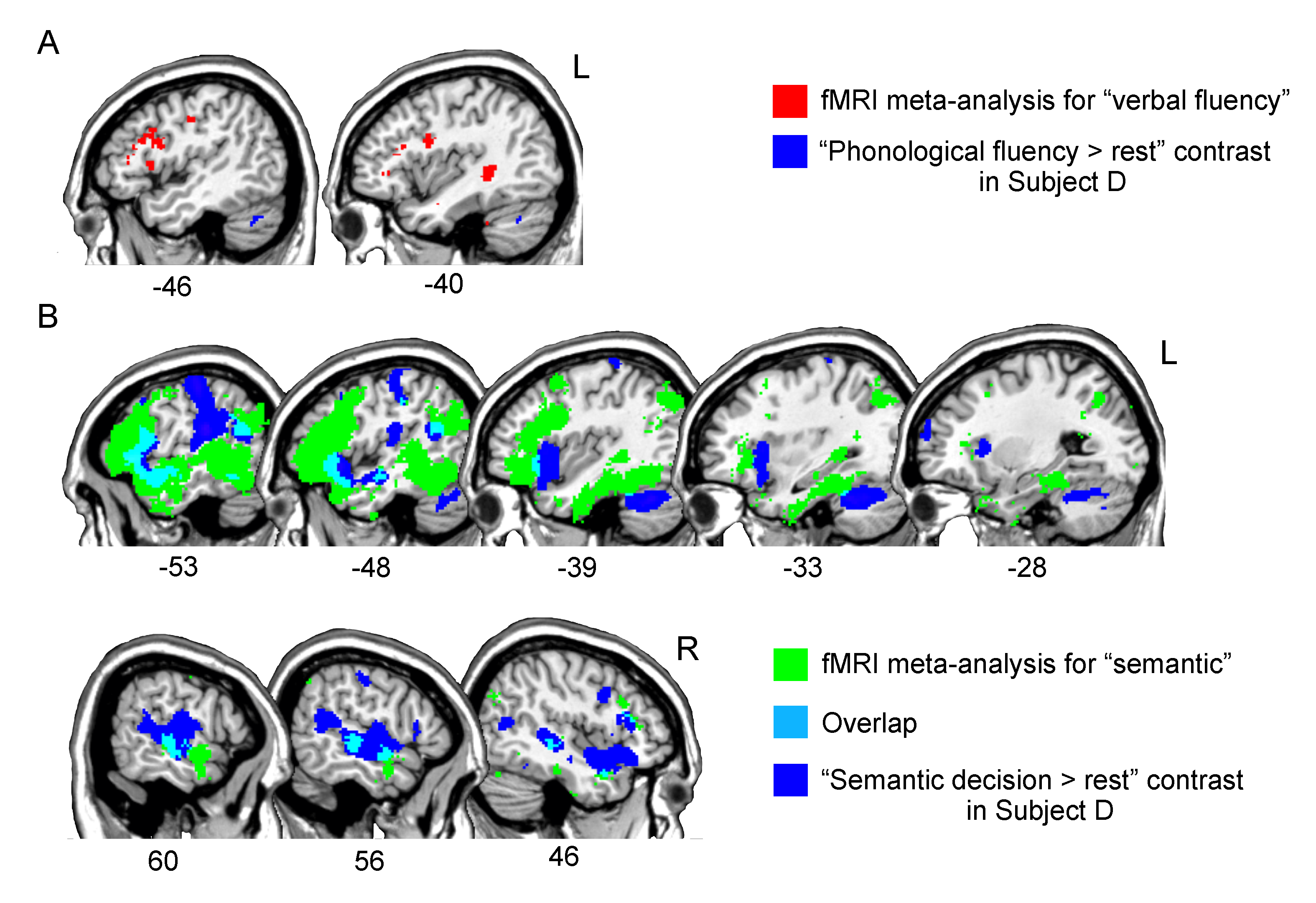

Supplement: Supplementary file 1 [file Image_1.TIF]
