# Supplementary material for: Developmental Dynamic Dysphasia: Are Bilateral Brain Abnormalities a Signature of Inefficient Neural Plasticity?
Source: Front Hum Neurosci. 2020 Mar 24;14:73. doi: 10.3389/fnhum.2020.00073 (PMC7107010; doi:10.3389/fnhum.2020.00073)
Supplement: Supplementary file 2 [file Table_1.docx]

**Supplementary Material**

**Brain activation during language and motor tasks compared to normative activations**

Methods: Since in the current study we did not have a control group for the fMRI experiment, the extent of overlap between the brain activation for the Phonological Fluency and Semantic Decision tasks in Subject D and fMRI-related activations resulting from a fMRI meta-analysis were examined using Neurosynth platform (automated meta-analysis of fMRI data. Yarkony et al. 2011). Neurosynth was used to perform a meta-analysis for the term “Semantic” and “Verbal Fluency”. A term-based search was performed resulting in 84 studied for the term “verbal fluency” and 1031 studies for the term “semantic” (search performed on April 10. 2019). Then, an uniformity test was generated for each search. The normalized results for the Phonological Fluency vs. rest were overlapped with the meta-analysis results for the term “verbal fluency”. The Semantic Decision vs. rest contrast in subject D was overlapped with the fMRI maps derived from the meta-analysis for the term “semantic”.

Results: First, no overlap between the statistical map derived from the meta-analysis of healthy subjects for the term “verbal fluency” and the activations of subject D during the Phonological Fluency task vs. Rest was found (Figure S1) using an uncorrected threshold of p < 0.001. The lack of overlap was expected since subject D showed very low activation for this task, in agreement with his poor performance in fluency tasks. Subject D showed an atypical pattern of activation involving the right inferior and middle frontal gyri (Figure 2A, Table 4), whereas the typical pattern of activations for fluency tasks in healthy subjects, as reflected in the map derived from the fMRI meta-analysis, involved mostly left hemisphere areas (i.e., inferior frontal gyrus, temporal cortex). This -absence of- overlap is illustrated in Figure S1A. Second, the meta-analysis of fMRI reporting activations associated to the term “semantic” revealed an extended bilateral network that overlapped to greater extent the fMRI activation map for the Semantic Decision vs. rest contrast in subject D (see Figure S1B).

**References**

Yarkoni, T., Poldrack, R. A., Nichols, T. E., Van Essen, D. C., and Wager, T. D. (2011). Large-scale automated synthesis of human functional neuroimaging Q21 data. Nat. Methods 8, 665–670. doi: 10.1038/nmeth.1635

**Parameters used in the TMS experiment**

Table S1. Stimulation left hemisphere for the hand (C3).

|  |  | **Motor threshold** | **MEP cortical latency (ms)** | **MEP cortical amplitude (microV)** |
| --- | --- | --- | --- | --- |
| **Rest** |  | 37 % |  |  |
|  | Right 1^st^ dorsal interosseus |  | 20.12 | 0.34 |
|  | Left 1^st^ dorsal interosseus |  | 19.2 | 0.44 |
|  | Right 1^st^ abductor hallucis |  | - | - |
|  | Left 1^st^ abductor hallucis |  | - | - |
| **Facilitation** |  |  |  |  |
|  | Right 1^st^ dorsal interosseus |  | 20.55 | 1.05 |
|  | Left 1^st^ dorsal interosseus |  | 20.40 | 0.7 |
|  | Right 1^st^ abductor hallucis |  | - | - |
|  | Left 1^st^ abductor hallucis |  | - | - |
|  |  | Silent period (mean)-ms |  |  |
|  | Right 1^st^ dorsal interosseus | 13.9 |  |  |
|  | Left 1^st^ dorsal interosseus | 15.7 |  |  |

Table S2. Stimulation right hemisphere for the hand (C4).

|  |  | **Motor threshold** | **MEP cortical latency (ms)** | **MEP cortical amplitude**  **(microV)** |
| --- | --- | --- | --- | --- |
| **Rest** |  | 60% |  |  |
|  | Right 1^st^ dorsal interosseus |  | 19.88 | 0.2 |
|  | Left 1^st^ dorsal interosseus |  | 21.72 | 0.22 |
|  | Right 1^st^ abductor hallucis |  | - | - |
|  | Left 1^st^ abductor hallucis |  | - | - |
| **Facilitation** |  |  |  |  |
|  | Right 1^st^ dorsal interosseus |  | 20.6 | 0.1 |
|  | Left 1^st^ dorsal interosseus |  | 21.3 | 0.3 |
|  | Right 1^st^ abductor hallucis |  | - | - |
|  | Left 1^st^ abductor hallucis |  | - | - |
|  |  | Silent period (mean -ms) |  |  |
|  | Right 1^st^ dorsal interosseus | 14.1 |  |  |
|  | Left 1^st^ dorsal interosseus | 4.85 |  |  |

Table S3. Stimulation left hemisphere for the legs (Cz- counterclockwise).

|  |  | **Motor threshold** | **MEP cortical latency (ms)** | **MEP cortical amplitude**  **(microV)** |
| --- | --- | --- | --- | --- |
| **Rest** |  | 35 % |  |  |
|  | Right 1^st^ dorsal interosseus |  | - | - |
|  | Left 1^st^ dorsal interosseus |  | - | - |
|  | Right 1^st^ abductor hallucis |  | 43.5 | 0.93 |
|  | Left 1^st^ abductor hallucis |  | 47 | 0.16 |
| **Facilitation** |  |  |  |  |
|  | Right 1^st^ dorsal interosseus |  | - | - |
|  | Left 1^st^ dorsal interosseus |  | - | - |
|  | Right 1^st^ abductor hallucis |  | 37.4 | 1.30 |
|  | Left 1^st^ abductor hallucis |  | 41.45 | 0.45 |
|  |  | Silent period (mean-ms) |  |  |
|  | Right 1^st^ abductor hallucis | 36.5 |  |  |
|  | Left 1^st^ abductor hallucis | 45.7 |  |  |

Table S4. Stimulation right hemisphere for the legs (Cz- clockwise).

|  |  | **Motor threshold** | **MEP cortical latency (ms)** | **MEP cortical amplitude**  **(microV)** |
| --- | --- | --- | --- | --- |
| **Rest** |  | 45 % |  |  |
|  | Right 1^st^ dorsal interosseus |  | - | - |
|  | Left 1^st^ dorsal interosseus |  | - | - |
|  | Right 1^st^ abductor hallucis |  | - | - |
|  | Left 1^st^ abductor hallucis |  | 41.12 | 0.32 |
| **Facilitation** |  |  |  |  |
|  | Right 1^st^ dorsal interosseus |  | - | - |
|  | Left 1^st^ dorsal interosseus |  | - | - |
|  | Right 1^st^ abductor hallucis |  | - | - |
|  | Left 1^st^ abductor hallucis |  | 39.15 | 1.15 |
|  |  | Silent period (mean-ms) |  |  |
|  | Right 1^st^ abductor hallucis | - |  |  |
|  | Left 1^st^ abductor hallucis | 57.8 |  |  |

The muscle condition during TMS was at rest and with weak voluntary contraction (20%) for the MEPs, and with strong voluntary contraction for the silent period.

**Figure Caption**

**Figure S1**. Overlap of subject D’s brain activation map during the language tasks (threshold: p < 0.001 uncorrected) with areas related to fluency and semantic functions in healthy subjects resulting from a fMRI meta-analysis with Neurosynth. **A.** Activation pattern resulting from the meta-analysis of activations associated to verbal fluency tasks is overlapped with the activation of subject D during the phonological fluency vs. rest contrast. Notice that there was no overlap since normally there is a left-lateralized inferior frontal and premotor activation in healthy subjects (red colour), and subject D showed a right lateralized activation as depicted in Figure 2. **B.** Activation resulting from the meta-analysis of activations associated to semantic tasks. No overlap with the activation of subject D during the semantic decision vs. rest contrast was found. L: left; R: right.
